# Analyzing comprehensive palatability of cheese products by multivariate regression to its subdomains

**DOI:** 10.1002/fsn3.48

**Published:** 2013-07-12

**Authors:** Kumiko Nakano, Yasushi Kyutoku, Minako Sawa, Shigenobu Matsumura, Ippeita Dan, Tohru Fushiki

**Affiliations:** 1 Laboratory of Nutrition Chemistry, Division of Food Science and Biotechnology, Graduate School of Agriculture, Kyoto UniversityOiwake-cho, Kitashirakawa, Sakyo-ku, Kyoto, 606-8502, Japan; 2 Functional Brain Science Laboratory, Jichi Medical University3311-1, Yakushiji, Shimotsuke, Tochigi, 329-0498, Japan; 3 Research and Development Initiatives, Chuo University1-13-27 Kasuga, Bunkyo-ku, Tokyo, 112-8551, Japan

**Keywords:** Culture, information, liking, preference, reward, sensory evaluation

## Abstract

The present study explored the possibility of generating a novel sensory evaluation instrument for describing comprehensive food palatability via its subdomains (rewarding, cultural, and informational) while keeping physiological factors constant. Seventy-five Japanese participants were asked to taste cheese samples and to respond to a questionnaire that was developed to dissect the distinct subdomains of palatability. The subsequent factor analyses revealed that three major factors may serve as distinct subdomains of palatability: rewarding, cultural, and informational, although the informational factor was not sufficiently robust. Multivariate regression analysis on cheese samples with exactly the same ingredients but sold in different packages led to different comprehensive palatability ratings due to the contribution of the cultural, but not the rewarding, factor. These results suggest that palatability is not merely determined by the physical and chemical properties that are intrinsic to a food product itself, but also depends on psychological properties that can arise through interaction between humans and the food product. The current study presents the first experimental demonstration that palatability could be dissociated to its subdomains.

## Introduction

**Figure 1:**
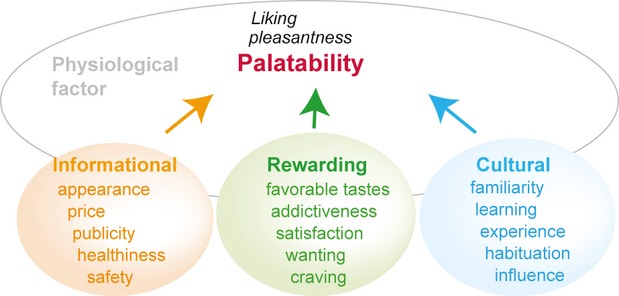
Hypothesized structure of palatability. Palatability is related to liking and preference. Among the putative subdomains of palatability, the most influential physiological factor is kept constant to focus on the effects of other factors. Informational, rewarding, and cultural factors are assessed in the current study.

In research on human food intake and acceptance, the term “palatability” has been used in its colloquial sense that reflects a positive hedonic evaluation under a given set of conditions, but its usage has not always been clear and consistent (Ramirez 1990). For example, in meat studies, consumers are often asked to evaluate meat-related products (e.g., steaks) using hedonic scales for tenderness, juiciness, flavor, and palatability, which is often substituted with overall liking and pleasantness (Mehaffey et al. 2009). Considering this ambiguity in terminology, in this article, we use the term palatability to represent the positive hedonic reward provided by foods. However, as past literature often uses compatible terms, including “liking” and “pleasantness,” to refer to palatability, we sometimes incorporate such inferences to discuss palatability (Fig. 1).

We should also note that palatability could either be a measure of food or of a person unless the source is specified. Ramirez (1990) pointed out three different views on palatability. The first classical view proposes that palatability is an objective property of foods (e.g., Kissileff 1986). This is consistent with our colloquial usage of the term that a certain food is more palatable than another: a palatable chocolate would be palatable to everybody. Conversely, in the second view, as far as food intake evokes the hedonic response of a human to sensory stimuli, the palatability should be regarded as a measure of the human (e.g., Le Magnen 1987). Accordingly, instead of saying that a food is palatable, we should specify that a food is palatable to any individual under certain defined conditions. In a sense, this is just a different side of the same coin: the former view focuses on the stimulus while the latter on the response. The third more holistic view involves the effects of learning and experiences (Ramirez 1990). Namely, the same chocolate would taste more palatable to one person than to another because they had undergone different chocolate experiences. From this perspective, palatability should not be regarded as a fixed food property intrinsic to a given food or an automatic physiological response, but rather as the context-dependent evaluation of a food by an individual (Blundell and Rogers 1991), which is largely influenced by experience (Mela 2006).

Although no individual has the same experiences with specific foods, some common factors affecting palatability seem present. If so, palatability may be dissected into componential subdomains, which in turn may allow its reconstruction with explicit descriptions of the contribution of each subdomain. To explore this possibility, we developed a questionnaire that would reflect the composite nature of palatability and explore major factors that represent distinct aspects of palatability. In our subsequent analysis of the questionnaire responses, we ascribed comprehensive food palatability to its subdomains using multivariate regression analyses. We will hereafter describe the theoretical background for this strategy.

Referring to a wealth of research on palatability and related food properties, we infer four factors: physiological, rewarding, cultural, and informational (Fig. 1). First, the physiological factor plays a pivotal role in determining palatability. Five basic tastes, sweetness, sourness, saltiness, bitterness, and umami (savoriness), elicit relatively fixed hedonic responses (Prescott 2001, 2004). For example, humans prefer sweetness and are averse to bitterness. These responses appear as early as from birth, and thereafter last throughout the lifetime (Steiner et al. 2001). Additionally, nutritional deficit affects palatability through a homeostatically driven motivational system. It has been shown that physical exercise that necessitates calorie consumption increases preference for sucrose (Horio and Kawamura 1998). When animals, including humans, detect deficient or imbalanced protein intake, a sparing of protein and a search for the deficient materials are initiated to maintain the necessary level of dietary protein intake (Mori et al. 1991). Thus, the alteration in the physiological state due to nutritional deficit, fatigue, and/or hunger can affect food palatability.

The second factor is the reward elicited by the intake of high-calorie foods. This has been well evidenced by studies on food craving, a strong desire to eat a particular food that may lead people to go out of their way to satisfy it (Zellner et al. 1999). Although the intake of high-fat content foods, sweets, carbohydrates/starches, and fat-containing fast foods (White et al. 2002) often induces excessive caloric consumption and leads to obesity, they are frequently preferred. Underlying the over consumption of high-calorie foods, animal studies have revealed the role of the reward system involving the dopaminergic and opiate systems in the brain (Imaizumi et al. 2001; Bello et al. 2011). Hence, the activation of the reward system by high-calorie foods may be the dominant factor that underlies food palatability.

Third, food is influenced by cultural factors established as part of the acquisition of culture, including beliefs, culinary traditions, and special occasions (Rozin 1996). For example, a recent implicit association experiment revealed that positive attitudes toward traditional diets relate to the type of breakfast eaten in childhood in young Japanese (Kimura et al. 2010). Also, elderly Italians' favorite foods are not only based on the sensory aspects of dishes but also on tradition and familiarity from youth (Laureati et al. 2006). A subject's current vegetable consumption is known to be predicted by their previous vegetable intake at home (Uglem et al. 2007), and another study demonstrated that the intake frequency of fruits and vegetables at home was positively associated with the intake of fruits and vegetables 5 years later (Larson et al. 2008). This evidence collectively suggests that food palatability is influenced by cultural factors, and past eating habits seem to be the most effective predictors.

Fourth, taste expectations formed based upon information can dramatically bias the sensory perception of food. Information such as the name of the item, its shape, and how it is packaged would have a great impact on forming expectations, which can either raise or lower liking ratings (Cardello et al. 1985; Rozin et al. 1998). Indeed, a series of experimental studies about the effects of information on food intake performed by Wansink and colleagues clearly demonstrated the importance of informational factors. For example, environmental cues including ambience, lighting, and sounds can create expectations and generate an intake bias. It is believed that expectations may lead a person to focus on particular aspects of taste that strengthen their initial expectations (Wansink and Park 2002; Wansink 2004). Environmental cues of food quality can take many forms, including price, labels, appearance, or names (Wansink et al. 2005). Moreover, it has been revealed that specific colors influence the perception of specific tastes, liking, and intensity ratings (Maga 1974; Johnson and Clydesdale 1982; Johnson et al. 1982; Zellner and Durlach 2003). Even names can influence the perception of unimodal basic tastes (Okamoto et al. 2009).

Among the four factors presented above, physiological is considered the most influential. However, this poses a serious experimental problem: individuals tend to attribute their own food intake to a highly influential physiological state such as hunger ignoring other important but less influential factors (Vartanian et al. 2008). Alteration in the physiological state due to nutritional deficit, fatigue, or hunger may lead to individual differences regardless of food. Thus, we decided not to pursue the obvious effects of physiological factors. Rather, we controlled to minimize the effect of physiological variance by making the time of day for the experiment and the temperature invariant. The remaining three factors were psychometrically assessed in reference to the overall palatability of a food sample.

Thus, we aimed to assess the feasibility of dissecting comprehensive palatability into the three componential subdomains. We developed a questionnaire that reflected the composite nature of palatability, and explored major factors representing its distinct aspects. In our subsequent analysis of the questionnaire responses, we ascribed comprehensive food palatability to its subdomains using multivariate regression analyses. Combining these, we explored the possibility of generating a novel analysis instrument for sensory evaluation.

## Materials and Methods

### Participants and procedure

Seventy-five Japanese participants (43 females, aged 19–79 years, median 20–39), with written informed consent, voluntarily participated.

To minimize physiological and physical interference, the experiment was conducted during off-meal hours (around 11:00 or 15:00) in a room set at 23°C. The absence of health issues, hunger, and satiety among participants was verified. Participants were asked to sit in front of a table, and to take three bites from one of three different types of cheese. Immediately after tasting a sample, participants were asked to respond to a questionnaire. Only one sample was tasted per day in a randomized order.

The study was approved by the institutional ethics committee of the Graduate School of Agriculture, Kyoto University.

### Food samples

Three commercially available cheeses (Cheeses A, B, and C) were sampled. Cheese A was a soft and natural Camembert cheese (Hokkaido Tokachi Camambert Kireteru, Meiji Co., Ltd., Tokyo, Japan). Cheeses B and C were processed cheeses accentuating natural flavor (B: Hokkaido Tokachi Smart Cheese; C: Hokkaido Tokachi 6P Cheese, Meiji Co., Ltd.); they were made of identical ingredients in the same ratios, but differed in the size of each piece, labeling, and wrapping design: this information was confirmed by the cheese manufacturer. In particular, Cheese B was thinner, had a more sophisticated wrapping design, and was more widely advertised on TV.

### Questionnaire

In the first part of the questionnaire, demographics including age, gender, hometown, existence of company to eat with, and physical conditions were measured.

Questionnaire items were sampled to suitably reflect three hypothetical subdomains of palatability (rewarding, cultural, and informational). First, using 5-point Likert type scales (1 = not at all, 5 = extremely), experts in nutrient chemistry and food research sampled items to explore their representation of the subdomains of palatability and compare their various perspectives. After the examination of content validity, 15 items were retained.

Of these 15 items, five were developed to measure the rewarding factor, which was measured by the degree of (1) desire caused by the addictiveness of a food, (2) level of difficulty in inhibiting urges to eat, (3) level of difficulty in inhibiting eating a food, (4) sense of satiety recognized by eating a food, and (5) sense of rewarding ingredients perceived by eating a food. Another five items were developed to measure the cultural factor, which was measured by the degree of (1) repeated exposure to a food, (2) dietary accustomedness to a food, (3) similarity with an accustomed food, (4) embeddedness of a food as a home-cooked taste, and (5) entrenched preference for a certain food. Finally, the remaining five items were developed to measure the informational factor, which comes from the (1) visual information from a food, (2) publicity of a food, (3) health information of a food, (4) perceived safeness of a food, and (5) perceived value for the price of a food (Table 1).

**Table 1:** Fifteen questionnaire items for the three componential factors of food palatability

a: Items putatively related to reward	
a1	Is the taste likely to be addictive to you?
a2	Does the taste make you feel compelled to pick up the food?
a3	Does the taste make you take another bite if you take a bite?
a4	Are you satisfied with the taste?
a5	Do you think the food tastes good because of rich fat sweetness or umami?
b: Items putatively related to culture	
b1	Are you used to the taste?
b2	Have you had a food that has the same or a similar taste to the food?
b3	Have you eaten food like this many times?
b4	Do you think your family (your parents, siblings, spouse, etc.) would like the taste of the food?
b5	Have you liked the taste of the food since your childhood?
c: Items putatively related to information	
c1	Does the food appear tasty?
c2	Have you ever seen this food in advertisements or heard of it by word-of-mouth?
c3	Have you ever heard anything good about the healthfulness of the food?
c4	Do you feel secure about the ingredients of the food?
c5	Do you think that the food seems expensive?

### Visual analog scales to measure comprehensive palatability

Comprehensive palatability for a cheese sample was measured in mm using 100-mm line visual analog scales (VAS) with descriptive anchors at each end (not palatable *extremely* for the left extremity, palatable *extremely* for the right extremity). VAS were used because their utility in measuring comprehensive palatability judgments was indicated in Prescott (2004).

### Psychometrical establishment of hypothetical subdomains of palatability (Cheese A data)

PASW statistics 19.0 was used throughout the analyses described hereafter. Data obtained for Cheese A were explored first. The psychometric adequacy of the items in the three hypothetical subdomains that reflect palatability was examined based on the classical testing theory (CTT; Nunnally and Bernstein 1994). Specifically, an exploratory factor analysis with a promax rotation was performed to categorize the 15 items. The criteria for extracting factors were based on (a) Kaiser's (1960) rule, (b) the scree test (Cattell 1966), and (c) interpretability of the extracted factors (Mertler and Vannatta 2005; Tabachnick and Fidell 2007). Stringent criteria for factor loadings at 0.45 were used based on criteria by Comrey and Lee (1992). After the extraction, the correlations among the factors were explored.

### Examination of unidimensionality of subdomains (Cheese B and C data)

Data obtained for Cheeses B and C were used in the subsequent analyses. The unidimensionality of the questionnaire for each sample was examined using parallel analysis where random data sets that contained the same number of items and participants as in the actual data sets were simulated to conduct an exploratory factor analysis. In order to confirm a unidimensionality, the first factor estimated from the observed data should be larger than that of the simulated data, and the subsequent factors estimated from the observed data should not be larger than those of the simulated data (Bernstein et al. 2007). The questionnaire was reexamined for Cheese B and C data. Criteria were set to be equal to those of Cheese A.

### Regression analyses and comparison of comprehensive palatability and its subdomains (Cheese B and C data)

A paired *t*-test (two-tailed) was performed to compare the VAS scores of comprehensive palatability between Cheeses B and C. In addition, paired *t*-tests (two-tailed, Bonferroni corrected) were performed to compare the rewarding and cultural subdomain scores for Cheeses B and C.

Upon establishing the unidimensionality of the subdomains, multiple regression analyses with backward elimination were, respectively, conducted for Cheeses B and C to explore whether the subdomains of palatability accounted for the comprehensive palatability.

## Results

### Psychometrical establishment of hypothetical subdomains of palatability (Cheese A data)

For Cheese A, an exploratory factor analysis with a promax rotation was performed on the 15 questionnaire items reflecting the three hypothetical subdomains of palatability. The three-factor structure could be extracted on Kaiser's rule and scree test. Ten items exhibited factor loading above 0.45 (Table 2). In the first factor, two items exhibiting excessively high interitem correlation were excluded. As a result, the remaining eight items retained the three-factor structure, and were, respectively, interpreted as “rewarding” (Cronbach's α = 0.88, *n*_items_ = 3), “cultural” (Cronbach's α = 0.82, *n*_items_ = 3), and “informational” (Cronbach's α = 0.20, *n*_items_ = 2) in accordance with the hypothesized subdomains of palatability. The factors, respectively, accounted for 23.5%, 22.3%, and 8.4% of variance in the items. Correlations between the first and the second factors, between first and the third, and between the second and the third were *r*(75) = 0.55, *P* < 0.01, *r*(75) = 0.28, *P* < 0.05, and *r*(75) = 0.24, *P* < 0.05, respectively. Although internal consistencies of the first two factors were sufficiently high, the third factor indicated a low internal consistency.

**Table 2:** Exploratory factor analysis with promax rotation for 15 items of Cheese A

		Factor loadings for food palatability		
		
	Communality (*h*^*2*^)	Rewarding	Cultural	Informational
a				
a1	0.838	0.920		
a2	0.887	**0.913**		
a3	0.885	0.929		
a4	0.792	**0.909**		
a5	0.468	**0.608**		
b				
b1	0.999		**1.032**	
b2	0.521		**0.625**	
b3	0.670		**0.625**	
b4	0.362			
b5	0.248			
c				
c1	0.238			
c2	0.358			**0.608**
c3	0.315			
c4	0.289			
c5	0.263			**0.466**

A criterion of 0.45 for factor loading was used as the cutoff for inclusion of items in a factor. Only factor loadings for items over the criterion are shown.

The factor loadings of the items finally included in the three factors are in bold.

### Examination of unidimensionality of subdomains (Cheese B and C data)

The parallel analysis indicated the unidimensionality of the rewarding and cultural factors, whereas it failed to detect unidimensionality of the informational factor. Multicollinearity among items or factors was absent. Internal consistency for the rewarding factor was 0.88 and 0.86 for Cheeses B and C, respectively, and for the cultural factor was 0.70 and 0.81, respectively. The internal consistencies for the first two factors were sufficiently high for Cheeses B and C, but were only 0.19 for Cheese B and 0.06 for Cheese C. These analyses led us to conclude that the rewarding and cultural factors are reliable subdomains, accounting for comprehensive palatability.

### Regression analyses and comparison of comprehensive palatability and its subdomains (Cheese B and C data)

Comprehensive palatability as measured by VAS was 65.6 ± 24.2 (mean ± standard deviation) for Cheese B and 56.2 ± 26.4 for Cheese C. A paired *t*-test revealed that comprehensive palatability was significantly higher for Cheese B (*t*(74) = 2.42, *P* < 0.05, *d*s = 0.37), despite the fact that the ingredients in both types of cheese were identical.

Scores for the rewarding and cultural subdomains averaged across items were 3.24 ± 1.07 and 3.25 ± 0.92, respectively, for Cheese B and 2.83 ± 1.01 and 3.00 ± 1.04, respectively, for Cheese C. Paired *t*-tests revealed that the rewarding factor score was significantly higher for Cheese B (*t*(74) = 2.37, *P* < 0.05, *d*s = 0.39). No significance was found for the cultural factor.

Accountability of the rewarding and cultural factors on comprehensive palatability was measured using VAS for Cheeses B and C, respectively, which were then examined using multiple regression with the backward elimination method. The resulting equation for each cheese is shown in Table 3. Although the accountability of the models was high for both types of cheese, there were striking differences: only the rewarding factor accounted for the palatability of Cheese B, while the cultural as well as rewarding factors accounted for the palatability of Cheese C (Table 3).

**Table 3:** Multiple regression analyses with backward elimination method to account for palatability of Cheeses B and C by subdomains

	Cheese B			Cheese C		
		
Predictor variable	*R*^2^	*β*	*F* model (df_1_, df_2_)	*R*^2^	*β*	*F* model (df_1_, df_2_)
Sequence 1	0.715		90.446* (2, 72)	0.688		79.296* (2, 72)
Rewarding		0.801*			0.740*	
Cultural		0.079			0.163*	
Sequence 2	0.711		179.428* (1, 73)			
Rewarding		0.843*				

*F* model df_1_, stands for degree of freedom for effect; df_2_, degree of freedom for error; *P < 0.05; Backward elimination was terminated at Sequence 1 for Cheese C.

Taken together, these differences in comprehensive palatability could be a reflection of a larger contribution of the cultural factor in Cheese C than in Cheese B in the resulting equations.

## Discussion

The current study explored the possibility of generating a novel sensory evaluation instrument for describing palatability. Although palatability has only been vaguely described as a single food attribute, the current study successfully dissected palatability into subdomains and quantitatively associated their relation, presenting a novel, quantitative approach for assessing food palatability.

### Subdomains of palatability

As exemplified in the proverb, “hunger is the best spice,” the most influential subdomain of palatability is obviously the physiological factor. However, the predominant influence of the physiological factor has prevented the decomposition of palatability, as the food intake of individuals tends to be more affected by a highly influential physiological state such as hunger than by other important but less influential factors (Vartanian et al. 2008). To overcome this issue, the current study employed a unique attempt to eliminate the possible effects of physiological factors by controlling the physiological states of the participants and focusing on the analyses of contributions of other less influential but important factors.

Consequently, a factor analysis, applied on the sensory evaluation of a cheese sample employing the 15 palatability-related items, extracted three factors as predicted. The subsequent adjustment processes of eliminating seemingly duplicated items and those with insufficient factor loading still yielded three factors, which were reasonably interpreted as rewarding, cultural, and informational, consisting of three, three, and two items, respectively.

We suggest that the rewarding and cultural factors are stable and reliable subdomains of palatability, and that although the third factor related to information may be present, further exploration is required to establish it as robust.

### Multivariate regression model for palatability

The results of the factor analyses and subsequent unidimensionality assessment of rewarding and cultural factor suggested the appropriateness of using the two-variable regression model to account for total palatability with its subdomains. The samples were both available commercial cheese products sold in different packages, with different names, serving sizes, and shapes, but they actually consisted of exactly the same ingredients. Participants were not informed of this fact. Use of these samples was expected to contrast out the relative importance of each palatability subdomain and their net contribution to the formulation of the total palatability.

Interestingly, although the samples were made of the same ingredients, the comprehensive palatability was significantly different. Comparison of rewarding and cultural factor scores between Cheeses B and C revealed that the rewarding factor score was significantly higher for Cheese B, while the cultural factor score was similar. Moreover, multiple regression analyses exhibited a predominant contribution of the rewarding factor in explaining the comprehensive palatability of Cheese B, while both factors were shown to be appropriate for Cheese C. We thus concluded that the greater comprehensive palatability of Cheese B was attributed to the greater contribution of the rewarding factor of Cheese B.

This observation clearly demonstrates that the subdomains of food palatability can have substantially large effects: so much so as to alter the total palatability of a food. To our knowledge, this is the first experimental demonstration quantifying the effects of food palatability subdomains and their contribution to the formation of comprehensive palatability.

The observed difference in the overall palatability and the cultural factor could be interpreted from the perspective of flavor preference conditioning, in which omnivorous animals including humans learn to prefer flavors that are associated with positive consequences (Yeomans 1998; Yeomans et al. 2004). Namely, Cheese C was perceived as more palatable because it was more associated with past eating experiences that had positive consequences. Actually, Cheese C is sold in a package that looks similar to the conventional processed cheese products in the Japanese market. Its product name is coherent enough to allow its inclusion in the conventional processed cheese category. Thus, Cheese C is likely to be perceived as an extension of conventional processed cheese products. On the other hand, Cheese B is intended to offer a more compact and slim package, and appears with a different product name, together emphasizing its convenient usage. Thus, this type of processed cheese product is new to the Japanese market, and Cheese B might be perceived less in association with past eating experiences of conventional processed cheese products compared with Cheese C.

## Conclusion

The current study presents the first experimental demonstration that food palatability can be dissected into its subdomains, which in turn can reconstitute comprehensive palatability with an explicit description of the contribution of each componential subdomain. Such a quantitative approach using a multivariate regression model would be effective in analyzing detailed aspects of palatability when designing and evaluating food products, and would provide a novel sensory evaluation instrument for describing palatability. As an intriguing future application, the multivariate model may be sensitive enough to quantitatively illustrate differences in palatability perception across age, gender, or generations, and thus would serve as a valuable tool in food product development.

## References

[b1] Bello NT, Patinkin ZW, Moran TH (2011). Opioidergic consequences of dietary-induced binge eating. Physiol. Behav.

[b2] Bernstein IH, Rush AJ, Carmody TJ, Woo A, Trivedi MH (2007). Clinical vs. self-report versions of the quick inventory of depressive symptomatology in a public sector sample. J. Psychiatr. Res.

[b3] Blundell JE, Rogers PJ (1991). Hunger, hedonics, and the control of satiation and satiety.

[b4] Cardello A, Maller O, Masor HB, Dubose C, Edelman B (1985). Role of consumer expectancies in the acceptance of novel foods. J. Food Sci.

[b5] Cattell RB (1966). The scree test for the number of factors. Multivar. Behav. Res.

[b6] Comrey AL, Lee HB (1992). A first course in factor analysis.

[b7] Horio T, Kawamura Y (1998). Influence of physical exercise on human preferences for various taste solutions. Chem. Senses.

[b8] Imaizumi M, Takeda M, Sawano S, Fushiki T (2001). Opioidergic contribution to conditioned place preference induced by corn oil in mice. Behav. Brain Res.

[b9] Johnson J, Clydesdale FM (1982). Perceived sweetness and redness in colored sucrose solutions. J. Food Sci.

[b10] Johnson J, Dzendolet E, Damon R, Sawyer M, Clydesdale FM (1982). Psychophysical relationships between perceived sweetness and color in cherry-flavored beverages. J. Food Prot.

[b11] Kaiser HF (1960). The application of electronic-computers to factor analysis. Educ. Psychol. Measur.

[b12] Kimura A, Wada Y, Ohshima K, Yamaguchi Y, Tsuzuki D, Oka T (2010). Eating habits in childhood relate to preference for traditional diets among young Japanese. Food Qual. Prefer.

[b13] Kissileff HR (1986). Quantitative relationship between palatability and food intake in man. Interact. Chem. Senses Nutr.

[b14] Larson NI, Neumark-Sztainer DR, Harnack LJ, Wall MM, Story MT, Eisenberg ME (2008). Fruit and vegetable intake correlates during the transition to young adulthood. Am. J. Prev. Med.

[b15] Laureati M, Pagliarini E, Calcinoni O, Bidoglio M (2006). Sensory acceptability of traditional food preparations by elderly people. Food Qual. Prefer.

[b16] Le Magnen J, Boakes RA, Popplewell DA, Burton MJ (1987). Palatability: concept, terminology, and mechanisms. Eating habits: food, physiology and learned behaviour.

[b17] Maga JA (1974). Influence of color on taste thresholds. Chem. Senses.

[b18] Mehaffey J, Brooks JC, Rathmann RJ, Alsup EM, Hutcheson JP, Nichols WT (2009). Effect of feeding zilpaterol hydrochloride to beef and calf-fed Holstein cattle on consumer palatability ratings. J. Anim. Sci.

[b19] Mela DJ (2006). Eating for pleasure or just wanting to eat? Reconsidering sensory hedonic responses as a driver of obesity. Appetite.

[b20] Mertler CA, Vannatta RA (2005). Advanced and multivariate statistical methods.

[b21] Mori M, Kawada T, Ono T, Torii K (1991). Taste preference and protein nutrition and L-amino acid homeostasis in male Sprague–Dawley rats. Physiol. Behav.

[b22] Nunnally JC, Bernstein IH (1994). Psychometric theory.

[b23] Okamoto M, Wada Y, Yamaguchi Y, Kimura A, Dan H, Masuda T (2009). Influences of food-name labels on perceived tastes. Chem. Senses.

[b24] Prescott J (2001). Taste hedonics and the role of umami. Food Aust.

[b25] Prescott J (2004). Effects of added glutamate on liking for novel food flavors. Appetite.

[b26] Ramirez I (1990). What do we mean when we say “palatable food”?. Appetite.

[b27] Rozin P (1996). Sociocultural influences on human food selection.

[b28] Rozin P, Dow S, Moscovitch M, Rajaram S (1998). What causes humans to begin and end a meal? A role for memory for what has been eaten, as evidenced by a study of multiple meal eating in amnesic patients. Psychol. Sci.

[b29] Steiner JE, Glaser D, Hawilo ME, Berridge KC (2001). Comparative expression of hedonic impact: affective reactions to taste by human infants and other primates. Neurosci. Biobehav. Rev.

[b30] Tabachnick BG, Fidell LS (2007). Using multivariate statistics.

[b31] Uglem S, Frolich W, Stea TH, Wandel M (2007). Correlates of vegetable consumption among young men in the Norwegian National Guard. Appetite.

[b32] Vartanian LR, Herman CP, Wansink B (2008). Are we aware of the external factors that influence our food intake?. Health Psychol.

[b33] Wansink B (2004). Environmental factors that increase the food intake and consumption volume of unknowing consumers. Annu. Rev. Nutr.

[b34] Wansink B, Park S (2002). Sensory suggestiveness and labeling: do soy labels bias taste?. J. Sen. Stud.

[b35] Wansink B, Ittersum K, Painter J (2005). How descriptive food names bias sensory perceptions in restaurants. Food Qual. Prefer.

[b36] White MA, Whisenhunt BL, Williamson DA, Greenway FL, Netemeyer RG (2002). Development and validation of the food-craving inventory. Obes. Res.

[b37] Yeomans MR (1998). Taste, palatability and the control of appetite. Proc. Nutr. Soc.

[b38] Yeomans MR, Blundell JE, Leshem M (2004). Palatability: response to nutritional need or need-free stimulation of appetite?. Br. J. Nutr.

[b39] Zellner DA, Durlach P (2003). Effect of color on expected and experienced refreshment, intensity, and liking of beverages. Am. J. Psychol.

[b40] Zellner DA, Garriga-Trillo A, Rohm E, Centeno S, Parker S (1999). Food liking and craving: a cross-cultural approach. Appetite.

